# Isoprenoids Production from Lipid-Extracted Microalgal Biomass Residues Using Engineered *E. coli*

**DOI:** 10.3390/molecules22060960

**Published:** 2017-06-09

**Authors:** Sumeng Wang, Jianming Yang

**Affiliations:** Key Lab of Applied Mycology, College of Life Sciences, Qingdao Agricultural University, Qingdao 266109, China; smwang001@126.com

**Keywords:** lipid extracted microalgae, isoprenoids, detoxification

## Abstract

Microalgae are recognized as a third generation feedstock for biofuel production due to their rapid growth rates and lignin-free characteristics. In this study, a lipid extracted microalgal biomass residues was used as the raw material to produce isoprene, α-pinene and β-pinene with an engineered *E. coli* strain. We adopted an optimal sulfuric acid hydrolysis method (1:7 ratio of solid to acid solution, 32% (*w/v*) concentration of sulfuric acid solution at 90 °C for 90 min) to efficiently convert holocellulose into glucose efficiently (6.37 g/L). Futhermore, we explored a novel detoxification strategy (phosphoric acid/calcium hydroxide) to remove inhibitors and notably acetic acid, furfural and 5-hydroxymethylfurfural (5-HMF) were reduced by 5.32%, different number given later 99.19% and 98.22%, respectively. Finally, the fermentation concentrations of isoprene (223.23 mg/L), α-pinene (382.21 μg/L) and β-pinene (17.4 mg/L) were achieved using the detoxified hydrolysate as the carbon source, equivalent to approximately 86.02%, 90.16% and 88.32% of those produced by the engineered *E. coli* strain fermented on pure glucose, respectively.

## 1. Introduction

As the simplest member of isoprenoids, isoprene is an important platform chemical which could be used for producing medicines, pesticides, fragrances, and especially synthetic rubber [1,2,3]. Its derivatives, α-pinene and β-pinene, therefore, have the potential to be used for aviation fuel production owing to their compact structures and reactive olefin functionality properties [4,5]. Currently, because of the structural complexity of isoprenoids, the reduced availability of fossil resources, and the rapid growth of microorganisms, there is potential for isoprenoids production using biosynthetic methods to replace chemical synthesis [4,5]. Nowadays, two biosynthesis pathways are used to produce isoprenoids: the MEP pathway used by many eubacteria, green algae, and the chloroplasts of higher plants, and the MVA pathway that mainly exists in eukaryotes, archaebacteria, and the cytosols of higher plants [4]. In this study, a heterologous MVA pathway was used to produce isoprene, α-pinene and β-pinene with suitably engineered *E. coli* strains (YJM25, YJM29, FHR-2, respectively). However, due to the bottleneck of feedstock availability, biotechnology need to be applied to isoprenoids production by using more economical resources.

Initially, starch (potato, wheat etc.) was used as the first generation feedstock to produce bioethanol and other biobased materials. However, large amounts of crops are consumed during the fermentation, which could lead to severe food shortages, especially in the developing countries [6]. Later, in the past two decades, second generation feedstocks, lignocellulosic materials (straw, wood and grass) have been explored in biofuel production since they are cheap, renewable and don’t have compete with food supplies. These second generation materials are not applied to commercially due to their low yield and high cost resulting from the necessary hydrolysis process [6,7]. Therefore, a third generation feedstock is required to both satisfy the demand for biofuel production on a large scale and maintain ecological balance at the same time. Currently, some researchers have proposed algae as an ideal alternative for biofuel production due to its rapid growth, a unicellular or simple multicellular structure, lignin-free properties and ready availability on Earth [8].

Hence, in this paper, we utilized the lipid extracted microalgal biomass residues (LMBRs) as the feedstock to biosynthesize isoprene and its derivatives (α-pinene and β-pinene) using the engineered *E. coli*. This began with the conversion of LMBRs into fermentable sugar, and then the microbial fermentation was performed to generate bio-based isoprenoids. However, as it is rather time- and money-consuming, the LMBRs were hydrolyzed with sulfuric acid rather than enzymatic hydrolysis. Finally, 6.37 g/L of glucose was achieved based on the following hydrolysis conditions: ratio of solid to acid (1:7), acid concentration (32%), hydrolysis temperature (90 °C), and hydrolysis time (90 min). Since fermentation inhibitors (weak acid, furfural, 5-HMF) were formed during the acid hydrolysis process [6], we adopted five different detoxification strategies with recombination to reduce the inhibition consequence on the fermentation [6,7,8,9,10]. Among five methods, the phosphoric acid/calcium hydroxide detoxification combination, the best detoxification approach, was applied to remove acetic acid, furfural and 5-HMF by about 55.32%, 99.19% and 98.22% respectively. Finally, 223.23 mg/L of isoprene, 382.21 μg/L of α-pinene and 17.4 mg/L of β-pinene were obtained from the engineered *E. coli* strain fermented on the detoxified hydrolysate of LMBRs, accounting for about 86.02%, 90.16% and 88.32%, respectively, of the isoprene, α-pinene and β-pinene production by the *E. coli* strain using pure glucose, respectively.

## 2. Results and Discussion

### 2.1. Chemical Composition of LMBRs

**Figure 1:**
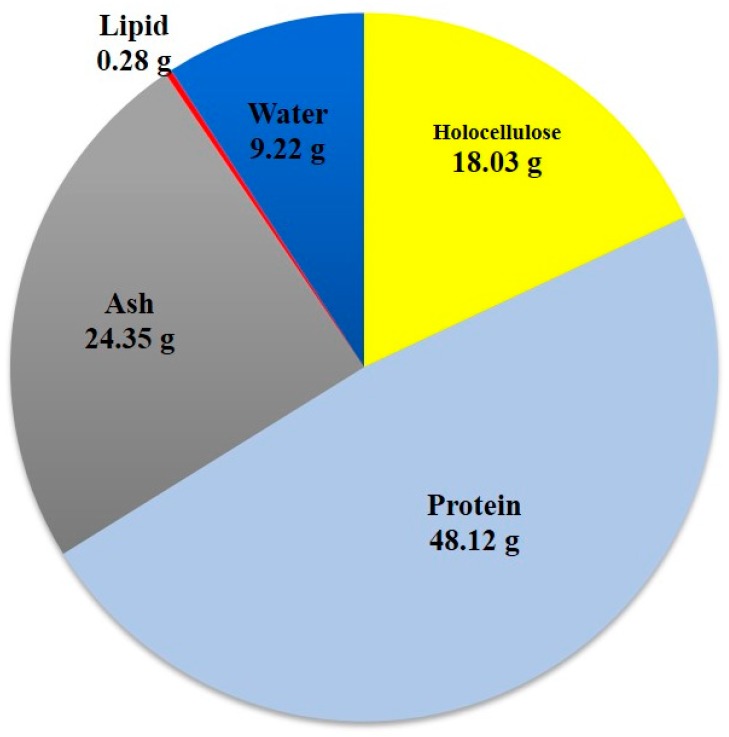
Chemical composition content of 100 g lipid extracted microalgae biomass.

As is shown in Figure 1, in a given 100 g amount of LMBRs, using appropriate quantification methods, there are about 18.03 g of holocellulose, 48.12 g of protein, 24.35 g of ash, 0.28 g of lipid and 9.22 g of water. Compared to the total lipid content (30–36 g) of microalgae in the previous study [11], this suggestes that the lipid in LMBRs has been almost completely extracted. What is more encouraging is that no lignin was found, as this represents most unfavorable threat to lignocellulose hydrolysis rejection, and therefore it must be removed prior to hydrolysis [12]. As we all know, to get rid of lignin during the process of pretreatment is rather costly and time-consuming as well. Hence, compared with lignocellulose (rice straw, switchgrass, etc.), LMBRs (with its absence of lignin) is considered an ideal alternative as the biofuel-producing material [13].

### 2.2. Optimization of Acid Hydrolysis Condition

**Figure 2:**
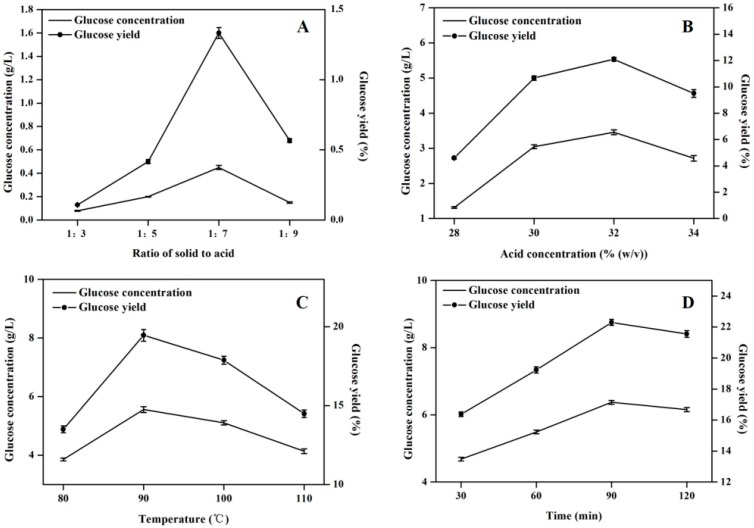
Effects of acid hydrolysis conditions on the glucose concentration and yield. (**A**) Effect of ratio of solid to acid on acid hydrolysis efficiency; (**B**) Effect of concentration of sulfuric acid on acid hydrolysis efficiency; (**C**) Effect of temperature on acid hydrolysis efficiency; (**D**) Effect of reaction time on acid hydrolysis efficiency. The experiment was performed in triplicate.

In this study, the “one-factor at-a-time” optimization strategy was applied to augment the hydrolytic efficiency by optimizing time, temperature, acid concentration and ratio of solid to acid solution respectively [14,15]. Figure 2 shows that the maximal glucose concentration (6.37 g/L) was obtained under the hydrolysis condition of 1:7 ratio of solid to acid solution, 32% (*w/v*) concentration of sulfuric acid solution at 90 °C for 90 min. The combined optimization effect could contribute to an approximately 142-fold increase in glucose concentration.

### 2.3. Inhibitor Changes with Different Detoxification Methods 

The acidolysis reaction could not only converts the lignocellulose into fermentable monomeric sugar, but some by-products (weak acid, furfural and 5-HMF, etc.) were also formed simultaneously. These by-products could inhibit the microorganism from producing high value-added products [16]. Due to absence of lignin in the LMBRs, only three main inhibitors including acetic acid, furfural and 5-HMF, would be produced during the acidolysis process. Acetic acid is formed primarily by hydrolysis of acetate groups of the acetate hemicellulose. While furfural and 5-HMF are derived from pentoses and hexoses, respectively [17].

**Figure 3:**
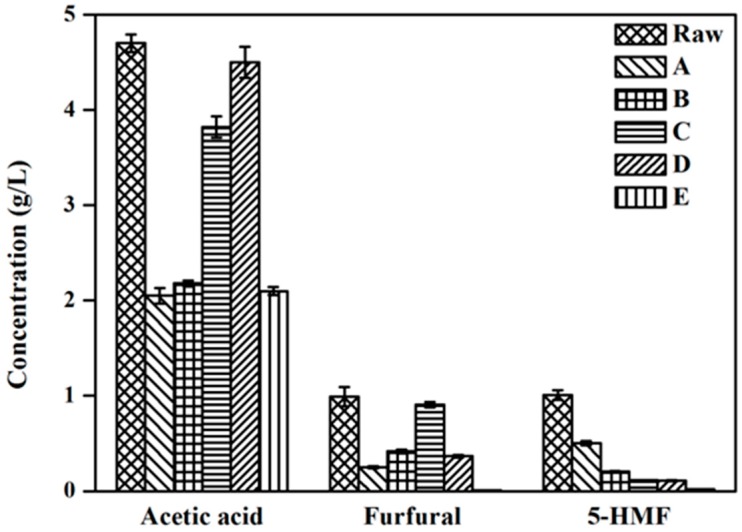
Inhibitors concentration of raw and detoxification hydrolysates with A–E five different detoxification strategies. These five detoxification methods are corresponding to the five methods A–E described in the Materials and Methods section, respectively. The experiments were performed in triplicate.

To date, many of investigations have shown that neutralization, overliming, activated charcoal, ion exchange resin and reducing agents all have the ability to remove the inhibitors from the acidolysis hydrolysate [6,7,8,9,10]. In the present study, we adopted five different kinds of detoxification strategies to explore the optimal detoxification method and analyzed the changes in the concentration of three types of inhibitors (acetic acid, furfural and 5-HMF). As is shown in Figure 3, compared with the raw hydrolysate, the concentration of all three types of inhibitors was reduced more or less in five different detoxification hydrolyzates.

Though methods A and B could decrease the acetic acid volume by more than half, the concentration of the furfural and 5-HMF inhibitors still remains too high. The hydrolysates after detoxification by methods C and D have a lower 5-HMF concentration, however, the acetic acid of the two hydrolysates was barely removed. Interestingly, in the detoxification hydrolysate using method E method, the acetic acid, furfural and 5-HMF were reduced about 55.32%, 99.19% and 98.22%, respectively. It showed that E detoxification method was the most efficient in the simultaneous removal of the three abovementioned inhibitors simultaneously. Meanwhile, compared with other detoxification methods, E method also presented a series of advantages such as lower cost, and easier operation in addition to the better capacity to remove inhibitory compounds. As a result, when cost, removal efficiency and difficulty of the detoxification process are all taken into consideration, method E has the highest potential to be used in the future industrialization field.

### 2.4. Effect of Detoxification on Isoprenoids Production

**Figure 4:**
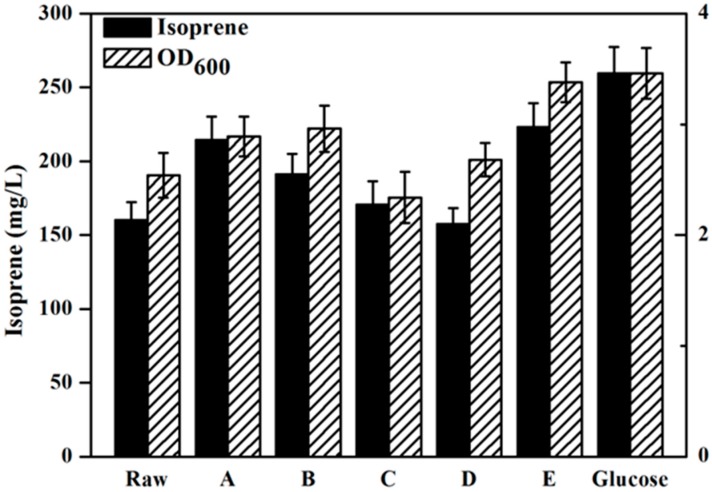
Isoprene production by the YJM25 strain using different carbon sources containing pure glucose, raw hydrolysate and five different kinds of hydrolysates detoxified by methods A–E. When OD_600_ reached ~0.6, cultures was induced at 30 °C for 24 h using 0.5 mM IPTG. The experiments were performed in triplicate.

**Figure 5:**
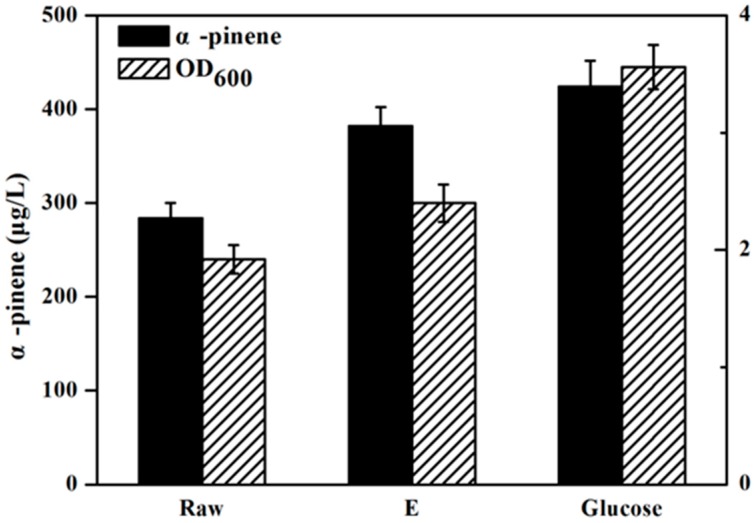
α-Pinene production by the YJM29 strain using different carbon sources containing pure glucose, raw hydrolysate and hydrolysate detoxified by method E. When OD_600_ reaches ~0.6, cultures were induced at 30 °C for 24 h using 0.5 mM IPTG. The experiments were performed in triplicate.

**Figure 6:**
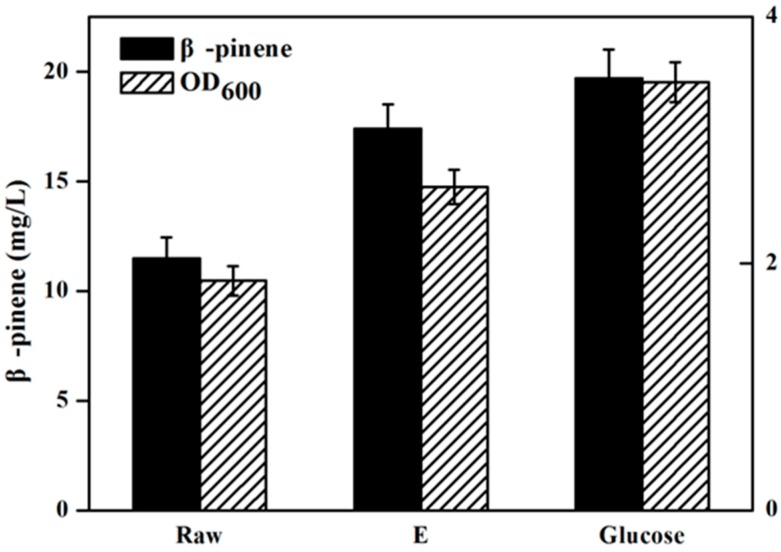
β-Pinene production by strain FHR-2 using different carbon sources containing pure glucose, raw and hydrolysate detoxified by method E. When OD_600_ reaches ~0.6, cultures were induced at 30 °C for 24 h using 0.5 mM IPTG. The experiments were performed in triplicate.

Figure 4 has presents the isoprene produced by the engineeried *E. coli* YJM25 with seven different carbon sources, including pure glucose, raw hydrolysate, and hydrolysates detoxified by methods A–E. As can be seen, compared with the raw hydrolysate fermentation (160.26 mg/L), isoprene yield produced by using group E hydrolysate (223.23 mg/L) was increased by about 40% after the E detoxification. The concentration of isoprene produced by group E hydrolysate represented 86.02% of that produced using pure glucose (259.52 mg/L). This percentage is much higher than those obtained using the other five types of hydrolysate (treatments A–D and raw). Therefore, the calcium hydroxide/phosphoric acid detoxification method was proved to be better than the other four kinds of detoxification methods for isoprene production with *E. coli*. As is expected, Figure 5 and Figure 6 have also demonstrated that, in comparision to raw hydrolysate fermentation, the yield of α-pinene and β-pinene were increased about 35% and 52%, respectively, when using hydrolysate detoxified by method E and α-pinene and β-pinene production accounted for about 90.16% and 88.32% of the yield produced on pure glucose. These results revealed that the *E. coli* fermentability was improved greatly after using the calcium hydroxide/phosphoric acid detoxification strategy.

Microorganism growth can be restricted by toxic inhibitive compounds such as furfurals, 5-HMF and organic acids [18]. Among those inhibitors, furfural was found to be able to inactivate the cell replication by breaking down the single-strand DNA [19,20,21,22]. Organic acids (acetic acid) derived from hemicellulose could cross the cell membrane, which resulted in a lower cell pH than normal and consequently inhibited cell activity [21,23]. Ultimately, the cell activity of *E. coli* was inhibited and this directly reduced its fermentation ability. As shown in Figure 3 and Figure 4, compared to other different carbon sources, the hydrolysate subjected to detoxification method E had the lowest concentration of inhibitors and consequently achieved the highest isoprene yield.

Chandra et al. have reported that although the toxicity of acetic acid on microorganisms is lower thanthat of furans (furfural and 5-HMF), the synergistic toxicity is possibly more severe when furans are present in conjunction with acetic acid [18]. In this study, we observed a similar result: as shown in Figure 3 and Figure 4, the amount of acetic acid in both hydrolysates detoxified by methods A and E was similar while that of the furans was higher in hydrolysate A than in hydrolysate E, which resulted in a lower isoprene production from hydrolysate A than with hydrolysate E, suggesting that the reason for the lower isoprene production from hydrolysate A might be a synergistic toxicity of acetic acid and furans.

## 3. Methods and Materials

### 3.1. Materials

LMBRs used in this study, which were the residual *Chlorella* biomass derived from oil extraction processes [11], were kindly provided by Prof. Tianzhong Liu (Qingdao Institute of Bioenergy and Bioprocess Technology, Chinese Academy of Sciences, Qingdao, China). Briefly, algae was mixed with ethanol at 1.5 MPa, 120 °C for 50 min and after cooling to room temperature, the residual algae and the oil solution was separated with centrifugation. Finally, the residual algae was collected to be used in this study. LMBRs were oven dried at 60 °C and milled to 60 mesh size. Sulfuric acid (H_2_SO_4_), sodium hydroxide (NaOH), calcium hydroxide (Ca(OH)_2_) and phosphoric acid (H_3_PO_4_) were bought from Sinopharm Chemical Reagent Co., Ltd. (Shanghai, China). Ion exchange resin (D310) was purchased from Tianjin Nankai University resin company (Tianjin, China). All of chemical reagents were analytical reagent grade.

### 3.2. Compositions Analysis

The compositions of LMBRs were determined using previously repoerted methods. Cellulose and hemicellulose were analyzed according to the method of NREL Laboratory Analytical Procedure (LAP) method [24]. In this study, LMBRs (300 mg) was treated with 72% sulfuric acid (3 mL) at 30 °C for 60 min. And then, deionized water (84 mL) was added into the mixture to dilute the sulfuric acid to a 4% concentration. The reaction mixture was incubated at 121 °C for 60 min. After cooling the mixture to room temperature, the residue was removed by filtration and the supernatant was collected and determined by HPLC to measure the monomeric sugar content including glucose, xylose, arabinose, galactose and mannose. The concentration of cellulose and hemicellulose were calculated according to the monomeric sugar content.

Total protein was measured based on the a previous study [25]. The nitrogen content was detected using an elemental analyzer (Elementar Vario EL III, Elementar Co., Hanau, Germany). Then, the crude protein concentration was calculated by the following formula:

protein concentration = nitrogen content × 6.25.

Lipid content was determined according to Lin Chen’s study using the gravimetric analysis method [11]. Dried algae (100 mg) was added into chloroform/methanol (1:2, *v*/*v*, 5 mL) and incubated at 65 °C for 1 h. After cooling the mixture to room temperature, the supernatant was separated from the LMBRs residue. This process was repeated three times. Then the three supernatants were combined into a vessel, the solution was dried and the lipid content was calculated by combining the dry weight of residue and the total LMBRs.

Ash analysis of LMBRs was measured according to the NREL Laboratory Analytical Procedure (LAP) [26]. LMBRs (1.0 g) was put in a crucible and incubated at 575 ± 25 °C for 24 h. After the sample and crucible were cooled to room temperature in a desiccator, they were weighed accurately. The ash content was determined according to the gravimetric method.

Organic solvent extractives (O.S.E) were analyzed by the TAPPI Standard Methods [27]. O.S.E of LMBRs was extracted by using a Soxhlet extraction apparatus, then the extractive was dried at 105 ± 3 °C to determine the O.S.E content was calculated according to the percentage of the extractive to unextracted LMBRs. The aqueous component was measured by the gravimetric method after drying the samples completely.

### 3.3. Optimization of the Acid Hydrolysis Process

Four kinds of factors that affect the acid hydrolysis were optimized with four kinds of factors including time, temperature, and concentration of sulfuric acid and the ratio of solid to acid. After lipid-extracting, the recovering microalgae residues was collected by filtration, then, the residues was washed with distilled water until the pH reached neutral and dried at 60 °C. The hydrolysis procedure was optimized as shown in Table 1. And the glucose concentration was detected and calculated by comparison with a standard using HPLC. All of experiments were repeated three times.

### 3.4. Inhibitor and Sugar Analysis of the Hydrolysates

Inhibitors and glucose were analyzed using HPLC equipped with a refractive index (RID) detector, and the concentration of inhibitors and glucose were calculated by converting peak areas to gram weights via their calibration curves. The HPX-87 Bio-Rad Aminex Column (300 mm × 7.8 mm, Bio-Rad, Hercules, CA, USA) was used for glucose detection. 0.005 M sulfuric acid was used as the mobile phase with a flow rate of 0.6 mL/min, and the column temperature was 55 °C. The concentrations of furfural and 5-HMF were determined with C-18 column (Nucleosil 100-5 C18, Merck, Darmstadt, Germany) with a gradient of 5–100% (*v/v*) methanol and 0.025% (*v/v*) of trifluoroacetic acid with a flow rate of 0.8 mL/min; formic acid and acetic acid were determined with AS11HC column which was eluted with 80% (*v/v*) water and 20% (*v/v*) of a mixture consisting of 0.4 mM NaOH and methanol (50% *v/v*) at a flow rate of 1.4 mL/min [6].

### 3.5. Detoxification

To produce isoprenoids with engineered *E. coli*, inhibitors that would be generated when the microalgae biomass is hydrolyzed with sulfuric acid should be removed first. As is shown in the following, five strategies have been developed to remove the inhibitors in the acidolysis hydrolysate based on previous studies with some modifications [6,7,8,9,10]:

- The hydrolysate was adjusted to pH 10 with sodium hydroxide, and then the solution was readjusted with sulfuric acid to pH 5. Anhydrous sodium sulphite (1 g/L) was added to the solution which was heated to 100 °C for 15 min. Then, 1% (*w/v*) activated carbon was mixed into the solution and incubated at 40 °C with shaking at 200 rpm for 1 h.
- The hydrolysate was neutralized with calcium hydroxide. After that, 1% (*w/v*) activated carbon was added into the solution and incubated at 40 °C with shaking at 200 rpm for 1 h.
- Sodium hydroxide was used to regulate the pH of the hydrolysate to 5.0, and then 1% (*w/v*) activated carbon was added into the solution and incubated 40 °C with shaking at 200 rpm for 1 h.
- Anion exchange resin (D301, Tianjin, China) was added to the hydrolysate with the loading concentration of 20% (*w/v*) until the pH reached 5.5. The mixed solution was kept at 24 °C, with shaking 200 rpm for 1 h.
- The pH of the hydrolysate was initially adjusted to 7.0 with calcium hydroxide, after that, the pH was readjusted to 5.5 with phosphoric acid.

All of the hydrolysated were filtered with a vacuum filter to obtain the supernatant after the inhibitors were removed with the different detoxification methods.

### 3.6. Biosynthesis and Analysis of Isoprenoids Produced Using the Engineered E. coli Strains

To verify whether the hydrolysate of LMBRs could be utilized to produce bio-based chemicals, several engineered *E. coli* strains were employed to produce isoprenoids. YJM25 (*E. coli* BL21^TM^ (DE3)/pYJM21, pYJM14), containing the optimized pathway and isoprene synthase, was used for isoprene fermentation [4]. YJM29 (*E. coli* BL21(DE3)/pYJM28), harboring a more efficient pathway for α-pinene production with the heterologous hybrid MVA pathway, GPPS2 and α-pinene synthase (Pt30), was utilized in α-pinene biosynthesis [5]. And FHR-2, including the *Artemisia annua* β-pinene synthase gene (QH6) and a heterologous hybrid mevalonate (MVA) pathway (*E. coli* BL21 (DE3) (pACYDuet-1-*mvaE*-*mvaS*-*GPPS2*-*QH6*, pTrcHis2B-*ERG8*-*ERG12*-*ERG19*-*IDI1*), was applied for β-pinene production [28]. The fermentation procedure was carried out as reported in the previous study [29] with some modifications. Shake-flask experiments were performed in triplicate using a series of sealed 600 mL shake flasks containing 100 mL of fermentation medium including glucose 2 g/L or a suitable concentration of acid hydrolysates. Optical density (OD) of the bacteria was measured with a spectrophotometer (Cary-50, Varian Inc., Palo Alto, CA, USA) at a wavelength of 600 nm. The isoprene, α-pinene and β-pinene production were analyzed as described earlier [4,5,28] by a gas chromatograph (GC) equipped with a flame ionization detector (FID) and a HP-1 column (30 m × 0.25 mm × 0.25 µm, Agilent, Palo Alto, CA, USA). Based on the retention time of standard samples, isoprene, α-pinene and β-pinene were identified, respectively. The concentration of target production was calculated from the peak area using a standard curve.

## 4. Conclusions

LMBRs are a potential raw material for biofuel production due to their absence of lignin and the presence of fermentable sugar in the microalgal residual biomass. 6.37 g/L glucose was achieved after hydrolyzing the lipid extracted microalgae with 1:7 ratio of solid to acid solution, 32% (*w*/*v*) concentration of sulfuric acid at 90 °C for 90 min. In order to increase the production of isoprene, α-pinene and β-pinene, inhibitors including acetic acid, furfural and 5-HMF of acid hydrolysate were removed by about 55.32%, 99.19% and 98.22%, respectively, by a new method (phosphoric acid/calcium hydroxide). Finally, 223.23 mg/L isoprene, 382.21 μg/L α-pinene and 17.4 mg/L β-pinene were produced, which representted about 86.02%, 90.16% and 88.32% of the quantities produced by pure glucose fermentation, respectively. Therefore, lipid extracted microalgae can be regarded as a promising material for the production of isoprenoids and other bio-based chemicals.

## Figures and Tables

**Table 1:** Exploration of the four kinds of hydrolysis condition was explored at four kinds of factors of LMBRs.

Optimization Factor	Range of Optimization Factor	Constant Factors
Ratio of solid to acid	1:3, 1:5, 1:7, 1:9	20% (*w/v*) concentration of sulfuric acid solution, at 80 °C for 30 min
Concentration of sulfuric acid	28% (*w/v*), 30% (*w/v*), 32% (*w/v*), 34% (*w/v*)	1:7 ratio of solid to acid solution, at 80 °C for 30 min
temperature	80 °C, 90 °C, 100 °C,110 °C	1:7 ratio of solid to acid solution and 32% (*w/v)* concentration of sulfuric acid solution for 30 min
Time	30 min, 60 min, 90 min, 120 min	1:7 ratio of solid to acid solution and 32% (*w/v*) concentration of sulfuric acid solution, at 90 °C
